# What is your diagnosis?

**DOI:** 10.4274/jtgga.galenos.2019.2019.0075

**Published:** 2020-06-08

**Authors:** Sultan Can, Fatih Aktoz

**Affiliations:** 1 Clinic of Obstetrics and Gynecology, Ağrı State Hospital, Ağrı, Turkey

A 27-year-old, gravida 3 para 1 woman who had pelvic pain was admitted to hospital. In her obstetric history, she had delivered a 1100 g female baby by cesarean section (C-section) one year ago because of acute fetal distress. A single fetus consistent with 11 weeks 5 days with no cardiac activity was detected in the uterus using ultrasonography. In addition, an arcuate uterus was observed. The patient was hospitalized for medical termination. The patient received a single dose of misoprostol 400 *μ*g vaginally. Six hours later, she had severe abdominal pain. Her blood pressure was 100/60 mmHg, and the pulse rate was 96 beats per minute. Her abdomen was distended with guarding and rebound tenderness. The patient also had chest pain. Transvaginal ultrasonography was performed and an intrauterine gestational sac was seen. A prior cesarean scar was intact and a 9 cm deep free fluid collection was observed in the perisplenic and perihepatic spaces. Her hemoglobin concentrations decreased from 9.4 g/dL to 7.8 g/dL within two hours. Emergency laparotomy was performed.

## Answer

**Figure 1:**
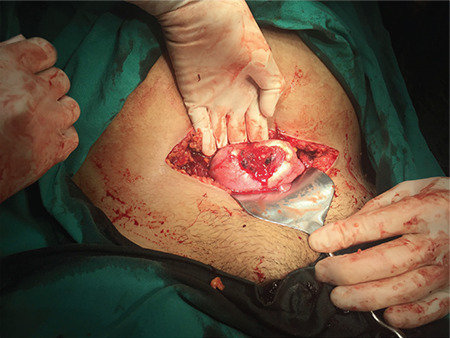
Intraoperative view of the uterine fundus rupture

Intraoperatively, uterine fundus rupture 4 cm in length was seen (Figure 1). The ruptured area was occupied by a clot and the gestational sac was still in the uterus and was successfully suctioned. Approximately 1000 mL blood collection was drained during the operation. The site of rupture was repaired using continuous 1-0 absorbable sutures. A single unit of packed red blood cells and one unit of fresh frozen plasma were transfused during laparotomy. An additional unit of red blood cells was given postoperatively. The patient was stable after the surgery and was discharged five days later.

There are guidelines for termination of pregnancy with misoprostol; however, there is no certain management on the route or dosage of misoprostol in patients with prior cesarean.

The International Federation of Gynecologists and Obstetricians recommends misoprostol use for missed abortion with a dose of 800 *μ*g vaginally every 3 hours before the 13^th^ week of gestation (1). Despite the lack of data about the safety of misoprostol, uterine rupture is one the major concerns for administration of the drug for termination of pregnancy especially for the second and third trimester. The incidence of rupture varies from about 1:1000 to 1:20,000 labors, and most of them were associated with a prior C-section (2). Management of uterine rupture is not certain because data on the management of uterine rupture in early pregnancy are limited and differ due to patient status. In general, hysterectomy is performed by reason of clinical condition. In the literature, there are some reports about conservative surgical repair of uterine rupture. An article by O’Connor and Gaughan (3) described pregnancies in patients in whom repair of a ruptured uterus had been performed previously. Seventeen of 18 pregnancies had a successful outcome and no cases of recurrent rupture were observed.

Cases of uterine rupture have been reported in early gestation even if small doses of misoprostol were given. For example, Jwarah and Greenhalf (4) and Kim et al. (2) reported two cases in 2000 and 2005, respectively. In both cases, uterine rupture was observed in first trimester of pregnancy following misoprostol use in women with prior C-sections. The case reported by Kim et al. (2) was interestingly similar to our case because uterine rupture existed in a horn and not the previous cesarean scar. In that case, it was said that the reason of uterine rupture could be misoprostol administration and local thin myometrium, not the previous caesarean scar. In 2006, Hidar wrote a letter to the editor about this case report and speculated about the possibility of ectopic pregnancy in the tubal interstitium (5). In view of the eccentric location of the gestational sac, the thin myometrium observed retrospectively by the authors, and the timing of pain after drug administration, this assessment seems reasonable.

This case seems to be the first report of a uterine fundus rupture occurring in the first trimester of gestation in a patient who was given a single vaginal dose of misoprostol.
